# Advances in Cancer Biology and Experimental Anticancer Therapies (2nd Edition)

**DOI:** 10.3390/biomedicines13123055

**Published:** 2025-12-11

**Authors:** Letizia Polito, Ewa Gajda

**Affiliations:** 1 Department of Medical and Surgical Sciences-DIMEC, General Pathology Section, Alma Mater Studiorum, University of Bologna, 40126 Bologna, Italy; 2 Department of Cell Biology and Immunology, Centre of Postgraduate Medical Education, 01-813 Warsaw, Poland; ewa.gajda@cmkp.edu.pl

Following the invitation from *Biomedicines*, we launched a second edition of the Special Issue “Advances in Cancer Biology and Experimental Anticancer Therapies”, as we believe it is truly important to continue focusing on advances in cancer biology and experimental antitumor therapies. Indeed, although chemotherapy, surgery, and radiotherapy remain the most commonly available cancer treatments, their use has shown numerous limitations, despite their demonstrated antitumor efficacy. The major limitations include the lack of selectivity for tumor cells, the development of drug resistance, and the risk of secondary tumor formation. Consequently, the study and development of alternative targeted therapies, such as immunotherapy and nanotherapy, have been extensively explored to find therapies with greater specificity for transformed cells and lower nonspecific toxicity. Therefore, it is essential to intensify studies on the molecular mechanisms of carcinogenesis and to develop new strategies for diagnosing and combating tumors.

This Special Issue includes eight manuscripts, among which there are six high-quality research articles, one review and one meta-analysis article, published from 2024 to 2025.

Acute lymphoblastic leukemia (ALL) is the most common cancer in children; however, racial and ethnic disparities persist in both the incidence and treatment outcome of ALL. Children of African descent have a significantly lower incidence of ALL compared to European Americans, while the disease is most common in Hispanic Americans [1]. The study by Mathew et al. investigated immune cell receptor expression across racial/ethnic populations and examined risk factors for relapse that could potentially influence outcomes in pediatric ALL. The authors proposed that altered expression of immune receptors in racially/ethnically and risk-stratified groups may provide insight into T cell- and NK cell-mediated immune surveillance against pediatric ALL [2].

Although several new therapeutic approaches have improved clinical outcomes in multiple myeloma, the majority of patients eventually experience disease relapse [3]. To address this, the study by Jiang et al., utilizing Mendelian randomization analysis, identified nine druggable genes, including Orosomucoid 1 and Oviductal Glycoprotein 1. The efficacy of two agonists of these molecules (pregnenolone for Orosomucoid 1 and irinotecan for Oviductal Glycoprotein 1) in suppressing myeloma cell growth suggests the potential application of these two molecules for clinical treatment of multiple myeloma [4].

In the study of Han et al. several druggable genes were evaluated for their association with prostate adenocarcinoma risk. The genes *MTHFD1* and *LGALS4* were identified as promising therapeutic targets for prostate adenocarcinoma, with evidence provided at multiomics levels. *LGALS4* was predominantly expressed in malignant cells and was correlated with enhanced immune checkpoint pathways and immunotherapy resistance. In contrast, *MTHFD1* was expressed in both tumor and microenvironmental cells and was associated with poor survival [5].

Single-cell RNA sequencing (scRNA-seq) technology has led to an exponential increase in the number of personalized data analyses in cancer patients. The clinical information obtained from single-cell analysis is crucial for exploring biomarkers of disease progression or treatment response and for guiding precise clinical decision-making for patients with malignant tumors [6]. Ma et al. carried out an integrative analysis of scRNA-seq data from lung adenocarcinoma patients. The results revealed that epithelial cells from stage IV tumors exhibited a distinct transcriptional profile compared to earlier stages. Several signaling pathways enriched in stage-specific crosstalk, including Wnt, PTN, and PDGF pathways, were identified, suggesting they may play critical roles in lung adenocarcinoma progression [7].

Hepcidin, a peptide synthesized by hepatocytes, is a crucial factor in systemic iron homeostasis of the organism. Since iron is essential for the highly active neoplastic cells, the induction of hepcidin has been implicated in the pathogenesis of anemia in patients with inflammatory and neoplastic diseases. It is well known that the IL-6/STAT3 signaling pathway is important for the transcriptional regulation of hepcidin [8]. Ferulic acid (FA) is an important active component of many traditional medicines that has a variety of biological activities, especially concerning oxidative stress and inflammation [9,10]. In the study by Al-Sanabra et al., the addition of FA to HepG2 cells significantly decreased the secretion levels of IL-6/STAT3 and hepcidin compared to the cells treated with LPS alone. The authors suggest that FA may be a potential therapeutic agent against hypoferremia and anemia resulting from dysregulated hepcidin levels in inflammatory and oncological diseases [11].

Sodium butyrate is a histone deacetylase inhibitor considered a promising anticancer drug for several types of cancer. It can inhibit the progression of prostate cancer, suppressing proliferation and inducing apoptosis [12]. Rutin is an antioxidant flavonoid found in many plants that has demonstrated antitumor activity in several cancer models [13]. The study by Alimudin et al. evaluated the effects of sodium butyrate and rutin combination therapy on metastatic castration-resistant prostate cancer cells. Enhanced apoptotic induction and elevated ROS levels were observed in combination-treated cells, alongside notable changes in cellular and nuclear morphology and mRNA expression [14].

The review by Lin et al. describes and summarizes some interesting oncotargets involved in the metabolic modulation of esophageal cancer, providing useful information for developing therapeutic strategies aimed at overcoming therapy resistance [15].

Tojjari et al. conducted a comprehensive meta-analysis of the clinical outcomes of chemoimmunotherapy in advanced biliary tract cancer [16]. The results demonstrated that chemoimmunotherapy significantly improved both overall survival and progression-free survival compared to chemotherapy alone in advanced biliary carcinoma, supporting the idea of chemoimmunotherapy as a first-line treatment strategy.

In conclusion, the findings from this Special Issue highlight the multifaceted potential of novel cancer therapy strategies that simultaneously target multiple cancer characteristics and improve conventional treatments.
